# Cognitive‐motivational, interpersonal, and behavioral functioning in relationship to treatment and research engagement in forensic patients with ADHD

**DOI:** 10.1002/jclp.23016

**Published:** 2020-07-13

**Authors:** Jenny A. B. M. Houtepen, Jelle J. Sijtsema, Rosalind Van der Lem, Anouk Scheres, Stefan Bogaerts

**Affiliations:** ^1^ Fivoor Research and Treatment Innovation Forensic Outpatient Center Rotterdam Rotterdam The Netherlands; ^2^ Department of Developmental Psychology, School of Social and Behavioral Sciences, Developmental Psychology Tilburg University Tilburg The Netherlands; ^3^ Department of Developmental Psychology Radboud University Radboud The Netherlands

**Keywords:** adult ADHD, forensic outpatients, research engagement, responsivity, treatment engagement

## Abstract

**Objectives:**

To provide more insight into treatment and research responsivity in offenders with attention‐deficit hyperactivity disorder (ADHD).

**Method:**

Via self‐reports and patients' scores on cognitive computer tasks, it was examined whether poorer cognitive‐motivational, interpersonal, and behavioral functioning were related to treatment no‐shows, longer treatment time duration intervals, and no‐show at the research appointment in 52 forensic outpatients with ADHD (*M*_age_ = 35.3, *SD* = 9.38). Treatment adherence was tracked for 10 appointments after research participation.

**Results:**

Regression analyses showed that higher self‐reported impulsivity was associated with research no‐show, and more alcohol use with longer treatment time intervals. Yet, self‐reported delay aversion was associated with fewer treatment no‐shows, and, uncontrolled for alcohol use, impulsivity was associated with shorter treatment time intervals in a subsample of patients.

**Conclusions:**

These preliminary results indicate that externalizing behaviors increase the risk for nonadherence in forensic ADHD patients, but that cognitive‐motivational problems also motivate patients to be more engaged.

## 1 INTRODUCTION

Attention‐deficit hyperactivity disorder (ADHD) is a developmental disorder (American Psychiatric Association, 2013) that persists fully or partially into adulthood in the majority of patients (Faraone, Biederman, & Mick, 2006). Adults with ADHD often have psychosocial impairments in multiple life domains (Goodman, 2007). Core ADHD symptoms and associated cognitive‐motivational deficits, including response inhibition difficulties and a need for direct stimulation, predispose patients to poor decision‐making (Mowinckel, Pedersen, Eilertsen, & Biele, 2015) and risk behaviors (Flory, Molina, Pelham, Gnagy, & Smith, 2006). In particular, patients with ADHD are at increased risk for offending. Compared to the general population, a 5 to 10‐fold increased prevalence of ADHD has been reported in forensic samples (e.g., Young, Moss, Sedgwick, Fridman, & Hodgkins, 2015). ADHD symptoms are further associated with an earlier age of onset for offending behavior and increased reoffending rates in forensic populations (Philipp‐Wiegmann et al., 2018; Young, Wells, & Gudjonsson, 2011). Furthermore, patients with ADHD are at an increased risk because of comorbid externalizing disorders (e.g., antisocial personality disorder, substance use disorders; Retz & Rösler, 2010), and associated risk factors, such as attachment problems (Houtepen, Sijtsema, Van der Lem, Van Hooydonk, & Bogaerts, 2019). Moreover, these risk factors are likely closely interlinked and interact throughout patients' lives. This makes offending in adults with ADHD a multifaceted problem for which adequate treatment is needed.

Yet, many adults with ADHD do not receive sufficient treatment (Kooij et al., 2010). ADHD in adulthood remains poorly recognized and underdiagnosed in clinical practice (Katzman, Bilkey, Chokka, Fallu, & Klassen, 2017). Next to pharmacological treatments, there are only a few evidence‐based treatment programs for adults with ADHD (e.g., Safren et al., 2010; Solanto et al., 2010), and in particular, psychological treatments that target ADHD and offending behavior are lacking. Only one forensic treatment program has been developed for patients with ADHD (Young & Cocallis, 2019). Yet, to date, its effectiveness has only been tested in nonforensic samples (Emilsson et al., 2011; Young et al., 2017).

In addition, recent research on forensic patients with ADHD suggests that when patients do receive treatment for offending, core ADHD symptoms and comorbid externalizing problems challenge patients' treatment adherence (Stoel, Houtepen, Van der Lem, Bogaerts, & Sijtsema, 2018). Although more research is warranted, these results suggest that the risk factors for which offenders with ADHD need help, are also the ones that may obstruct their way to recovery. Moreover, risk factors that are known to complicate treatment in difficult patient samples, often also complicate the conduct of research on these samples (Paige & Mansell, 2013), their treatment, and issues with responsivity. These research challenges are reflected in high research attrition rates among patients with ADHD and severe psychosocial and behavioral problems (e.g., Rich et al., 2014). Difficulties with conducting research may thus further explain the lack of knowledgeontreatment in forensic patients with ADHD. Although previous studies on treatment for ADHD indeed report high drop‐out rates in patients (e.g., Buitelaar, Posthumus, Bijlenga, & Buitelaar, 2019; Rich et al., 2014; Young et al., 2017), few studies have investigated risk factors for this poor treatment and research adherence in forensic patients with ADHD.

In the current study, we examine this responsivity issue in treatment and research in offenders with ADHD, by testing associations between patients' cognitive‐motivational, interpersonal, and behavioral functioning in relationship to treatment adherence and related issues. Previous research on these patients (Stoel et al., 2018; Woicik, Sijtsema, Van der Lem, & Bogaerts, 2017), focused mainly on patients' general and comorbid psychopathological symptoms (i.e., behavioral functioning; see below). Additionally, treatment characteristics were examined using retrospective research designs, which complicated the interpretation of findings. Using a prospective design to measure treatment adherence, we aim to replicate and extend previous results and examine symptoms underlying, and associated difficulties related to ADHD and offending.

### 1.1 Cognitive‐motivational functioning

Impairment in response inhibition (Barkley, 1997) and motivational “deficits” characterized by a heightened sensitivity for immediate rewards (Sonuga‐Barke, 2003) are among the most important deficits associated with patient variability in ADHD symptoms (Ma, van Duijvenvoorde, & Scheres, 2016; Sonuga‐Barke, Sergeant, Nigg, & Willcutt, 2008). Impulsive behavior resulting from response inhibition deficits is thought to result from difficulties in suppressing or interrupting (inappropriate) dominant behavioral responses in individuals with ADHD (Barkley, 1997). Deficits in response inhibition further affect poor cognitive, verbal, and emotional impulse control and result in difficulties with delaying gratification (Barkley, 2010). In contrast, motivational deficits primarily drive impulsive behavior on a cognitive and emotional level. Patients with ADHD behave impulsively because they discount the value of future rewards (i.e., temporal reward discounting; Jackson & MacKillop, 2016), or feel distressed when they have to wait for future rewards and therefore, are motivated to avoid delays (i.e., delay aversion; Sonuga‐Barke, 2003). There is some support that poor cognitive‐motivational functioning is a risk factor for offending in adults with ADHD: Both types of impulsivity have been associated with offending behaviors in adults with ADHD (e.g., McDonagh, Travers, & Bramham, 2018; Thorell, Sjöwall, Mies, & Scheres, 2017). Also, a number of studies comparing adult offenders with ADHD and nonoffending adults with ADHD, showed that offenders with ADHD had more inhibition problems (Bramham & Giollabhui, 2016; Ginsberg, Hirvikoski, & Lindefors, 2010; Meier, Perrig, & Koenig, 2012). These studies suggest that poor cognitive‐motivational functioning is a problem in forensic patients with ADHD.

Regarding responsivity to treatment, it can be hypothesized that cognitive‐motivational deficits associated with ADHD and offending also challenge treatment adherence in forensic patients with ADHD. In particular, because these deficits are expected to impact patients' abilities to commit to longer‐term goals, such as completing psychological treatmentto achieve better functioning in the long‐term. Yet, to date, this has not been examined in patients with ADHD. Most studies focusing on the role of cognitive‐motivational deficits in treatment have been conducted in patients with substance use disorders (e.g., Stevens et al., 2014), and only a few studies have been conducted in forensic populations (Fishbein et al., 2009; Peters, Petry, LaPaglia, Reynolds, & Carroll, 2013; Smeijers, Bulten, Buitelaar, & Verkes, 2017). Results of these studies are mixed. In some studies, response inhibition deficits were increased in patients who dropped‐out of treatment, and negatively related to progress as indicated by clinical professionals (Fishbein et al., 2009; Vergara‐Moragues et al., 2017). Yet, in other studies no associations with treatment drop‐out were found (Smeijers et al., 2017; Stevens et al., 2014). Also, (fewer) motivational deficits influenced substance abstinence during treatment in some substance‐abusing patient samples (Stevens et al., 2014), but this has not been supported in forensic patients (Peters et al., 2013).

Still, the increasing support for associations between cognitive‐motivational functioning and offending and treatment adherence in general, indicates that research on these links in forensic patients with ADHD is warranted. In particular, given the heterogeneity in cognitive‐motivational impairments in patients with ADHD (Ma et al., 2016; Sonuga‐Barke et al., 2008), examining combined effects of response inhibition and motivational deficits can be important to explain variability in treatment and research responsivity.

### 1.2 Interpersonal functioning

Next to personal characteristics, interpersonal issues, such as early family characteristics, are important in explaining differential outcomes in functioning in patients with ADHD (Hechtman, 1991; Sonuga‐Barke, Auerbach, Campbell, Daley, & Thompson, 2005). Research in adults with ADHD reported interpersonal issues such as having fewer friendships, more marital difficulties, and family dysfunction compared to individuals without ADHD (Eakin et al., 2004; Young, Toone, & Tyson, 2003). These interpersonal difficulties can disrupt the forming of secure attachment relationships in individuals with ADHD and subsequently may have an impact on adaptive functioning throughout the lifespan (Bowlby, 1973). Indeed, higher levels of insecure attachment have been reported in both children and adults with ADHD (Storebø, Rasmussen, & Simonsen, 2016).

In research on adult attachment and its outcomes, generally four styles of attachment are examined (Bartholomew & Horowitz, 1991). These styles are based on a dichotomized view of other people as being supportive, and the self as being worthy of this support, as described by Bowlby (1973). Hence, *securely* attached individuals are believed to have positive images of themselves and other people, whereas *preoccupied* individuals only have positive images of others, and negative images of the self. In contrast, *fearful‐avoidant* individuals have negative images of both self and other people, and *dismissive‐avoidant* attached individuals only have negative views of others. Recently, these insecure attachment styles were found to be elevated in a subsample of the current study, compared to healthy controls and associated with self‐reported externalizing behaviors as well (Houtepen et al., 2019). Similarly, insecure attachment styles (i.e., avoidant styles, in particular) have been considered important risk factors for offending in other clinical and offender samples too (Ogilvie, Newman, Todd, & Peck, 2014).

Regarding treatment responsivity, both insecure attachment and issues within patients' social environment have been found to impact upon the way in which patients are able to profit from psychological treatment (e.g., Feitsma, Popping, & Jansen, 2012; Levy, Ellison, Scott, & Bernecker, 2011). Research on attachment styles shows that individuals with insecure attachment are more likely to miss treatment appointments in primary care (Ciechanowski et al., 2006), and have more difficulty with forming a healthy therapeutic alliance because of distrust in others (Berry & Danguah, 2016). In turn, the quality of the therapeutic alliance has been strongly associated with treatment outcomes (Kozar & Day, 2012; Martin, Garske, & Davis, 2000). Furthermore, there is some indication that social support from family members is a protective factor against no‐show in forensic treatment (Feitsma et al., 2012; Sung, Belenko, Feng, & Tabacknick, 2004). Hence, it may be argued that because patients with ADHD often have lifelong social difficulties, they may have few prosocial individuals within their social networks (Garcia et al., 2018), who stimulate treatment engagement. As such, both insecure attachment styles and poor social support may be risk factors for poor treatment adherence in forensic patients with ADHD.

### 1.3 Behavioral functioning

Finally, next to several traditional background characteristics (e.g., young age, male gender, unemployment, forensic history, no prior treatment history, poor motivation to change; Cullen, Soria, Clarke, Dean, & Fahy, 2011; Fenger, Mortensen, Poulsen, & Lau, 2011; O'Brien, Fahmy, & Singh, 2009), one of the most reported risk factors for the treatment of nonadherence is the presence of externalizing behavioral problems. Despite some minor differences, studies consistently reported that patients with antisocial personality disorder, violent behavior (Cullen et al., 2011), substance abuse (Fenger et al., 2011; Matas, Staley, & Griffin, 1992), and psychopathy (Cullen et al., 2011) are at increased risk for treatment no‐show and drop‐out. Similarly, in earlier research on treatment no‐show in forensic outpatients with ADHD (Stoel et al., 2018), increased levels of antisocial behavior were associated with higher no‐show rates. Comorbidity rates with externalizing problems are increased in (forensic) patients with ADHD (Ginsberg et al., 2010; Retz & Rösler, 2010). Comorbidity with externalizing problems during childhood, increases risk for patients with ADHD to develop antisocial personality disorder later in life, and to engage in offending during adulthood (e.g., Storebø & Simonsen, 2016). Furthermore, comorbid substance use disorders are known risk factors for offending in patients with ADHD (Retz & Rösler, 2009). In adults with ADHD, substance use disorders, and (both clusters B and C) personality disorders are among the most commonly reported comorbidities (e.g., Katzman et al., 2017; Sobanski, 2006). Therefore, we also examined comorbid externalizing problems as a risk factor for poor treatment and research engagement in the current study.

### 1.4 The current study

In sum, treatment adherence may be challenging for offenders with ADHD (Stoel et al., 2018; Woicik et al., 2017), which can result in high no‐show and drop‐out rates during treatment. No‐shows and drop‐out in treatment results in high economic costs, and a waste of professional time (Moore, Wilson‐Witherspoon, & Probst, 2001). Moreover, poor treatment adherence may result in poorer treatment outcomes. In forensic psychiatry, where treatment goals not only focus on enhancing patient's mental health but also on reducing the risk for reoffending, poor adherence may thus also be associated with higher recidivism rates in non‐adherent patients (O'Brien & Daffern, 2015), hereby increasing the risk for unsafety within society. More knowledgeofrisk factors associated with treatment adherence in forensic patients with ADHD is thus important.

We examined patients' treatment adherence during forensic outpatient treatment, by examining associations between cognitive‐motivational, interpersonal, and (externalizing) behavioral functioning in relationship to no‐show and the time duration in days it took patients to finish a fixed number of treatment appointments (in the following: “treatment time intervals”). In addition, we examined no‐shows on research appointments. We hypothesized that poorer cognitive‐motivational (response inhibition and motivational deficits), interpersonal (insecure attachment and poor social support), and behavioral functioning (i.e., more externalizing behavior), were positively associated with no‐shows and longer treatment time intervals in forensic outpatients with ADHD. Finally, we examined whether these associations explained treatment and research non‐adherence above and beyond traditional demographic and background risk factors.

## 2 METHOD

### 2.1 Participants

Fifty‐two Dutch forensic outpatients with ADHD (*M*_age_ = 35.3, *SD* = 9.38, range = 19–61) participated in the study. Patients were recruited from a forensic outpatient center in The Netherlands in which a multimodal treatment program for ADHD and offending has been initiated. In this program, adults with ADHD receive treatment for their psychiatric disorder(s) and related offending behavior in different phases with the main goal of reducing risk for (re)offending. Patients receive compulsory treatment as part of a juridical measure, or are in treatment voluntarily after referral by a general practitioner or other mental health professional. Treatment phases include diagnostics, followed by psychoeducation for ADHD (Kooij et al., 2010) and its relationship with externalizing behavior, cognitive‐behavioral therapy for aggressive or other delinquent behavior (Hoogsteder & Bogaerts, 2018), and schema‐focused therapy (Young, 1999; Young, Klosko, & Weishaar, 2003) targeting personality problems if indicated. Additionally, patients receive ‘side modules’ including pharmacotherapy, practical support for social‐, financial‐, work related‐, or daily routine‐problems, and treatment for substance abuse if applicable. In the treatment program, the diagnostics phase usually includes psychological assessment by administration of the Diagnostic Interview for Adults with ADHD (DIVA; Kooij & Francken, 2010) and a psychiatric evaluation. The administration of the DIVA is conducted in the presence of older family members whenever possibleto reduce recall bias. In some cases, patients already received psychological assessment of ADHD in adulthood in another institution. These patients only receive a psychiatric interview to confirm the diagnosis when entering the ADHD treatment program here. The program is certified by the Foundation for Top Clinical Mental Health Care, and in line with both the Risk–Need–Responsivity model (Andrews, Bonta, & Hoge, 1990) and the European consensus statement on the treatment of Adult ADHD (Kooij et al., 2010).

In this study, most patients with ADHD (84.6%) had comorbid psychiatric disorder(s). Of these, substance use disorders (*n* = 27) and other impulse control disorders (*n* = 16) were most common. Also, three patients had a comorbid autism spectrum disorder, and two patients had a mild intellectual disability. Furthermore, 10 patients (19.2%) were diagnosed with cluster B personality disorder and 12 others (23.7%) with cluster B personality traits. Only 14 patients (26.9%) were currently receiving court‐ordered treatment, others were in treatment voluntarily. Of the 38 patients receiving treatment voluntarily, 22 (57.9%) did have a judicial past.

Of note, due to difficulties with including patients in the study and subsequent power issues, the current study also made use of data from 11 patients included in a pilot study (see Section 2.2). Group comparisons of the two patient groups using independent sample *t* tests and Mann‐Whitney *U* tests showed no differences on any of the study variables of interest. Hence, in general, there was variation in the type of treatment that patients received when they were included. Fourteen patients had (almost) finished the diagnostic phase, 23 patients were receiving psychoeducation, five patients received aggression‐regulation therapy, and two patients received schema‐focused therapy. Moreover, four patients were receiving long‐term maintenance therapy to keep treatment progress stabilized, and four patients were on a waiting list for receiving treatment within the ADHD program, or only received treatment side modules at the time. Half of the participants received psychotropic medication for ADHD and/or comorbid disorders.

### 2.2 Procedure

This study was conducted in accordance with the American Psychological Association's ethical guidelines and approved by the Ethical Review Board at the first author's university (EC‐2015.38).Beforedata collection, we conducted a power analysis using G*Power3 (Faul, Erdfelder, Lang, & Buchner, 2007) to determine the minimum number of participants needed to test multivariate associations of large effect sizes (*f* ^2^ = 0.35) with *α* = .05, in a regression model with six predictors. Results showed that a total sample of 46 participants was required to achieve a power of .80.

Between October 1, 2016 and December 31, 2018, patient inflow in the ADHD treatment program was tracked via electronic patient files and weekly team meetings for practitioners, in which all new patients are discussed. Inclusion criteria were male gender, being 18 years or older, and having an ADHD diagnosis. When these criteria were met, and there were no major objections for participation (such as being in crisis), therapists were asked to invite patients to participate after they (had almost) finished the diagnostic phase, or had just started treatment in the ADHD program. Patients who were interested received an information letter about the study's aim and procedure and were contacted by telephone to plan a research appointment at the outpatient center. Patients were informed that participation was voluntarily and that they could withdraw from the study at any time, without providing a reason for this. Participation included one appointment of approximately 2 h, including a short break.

During the research appointment, patients first signed written informed consent. Thereafter, patients participated in three computer tasks (i.e., a Go/No‐Go task, a temporal discounting task, and the Balloon Analogue Risk Taking task (BART; Lejuez et al., 2002; which was not used in the current study) and filled out a number of self‐report questionnaires together with one of the researchers. Of note, we did not provide any instructions for medication use on the day of the research appointment. Yet, patients were asked whether they used their medication on the day of the study visit, which only 10 patients did. These patients did not differ on their computer task scores from the other patients.

After the research appointment, patients' treatment adherence was followed for the first 10 treatment appointments that were planned at the outpatient center, via the electronic patient files. Patients received a gift voucher for their participation of either 5, 10, or 15 euros based on their performance on the BART. For a detailed explanation of this task, please see Lejuez et al. (2002). In contrast to the task described in Lejuez et al. (2002), in the task used in the current study, participants earned a number of points instead of a number of cents for every balloon that did not explode (i.e., the number of points equaled the number of clicks that they used to inflate the balloon). Based on the maximum amount of points that could be earned on the task, we divided all scores into a “low,” “medium,” and “high” scoring category and payed participants accordingly. Travelling expenses were reimbursed with an additional gift voucher.

**Figure 1:**
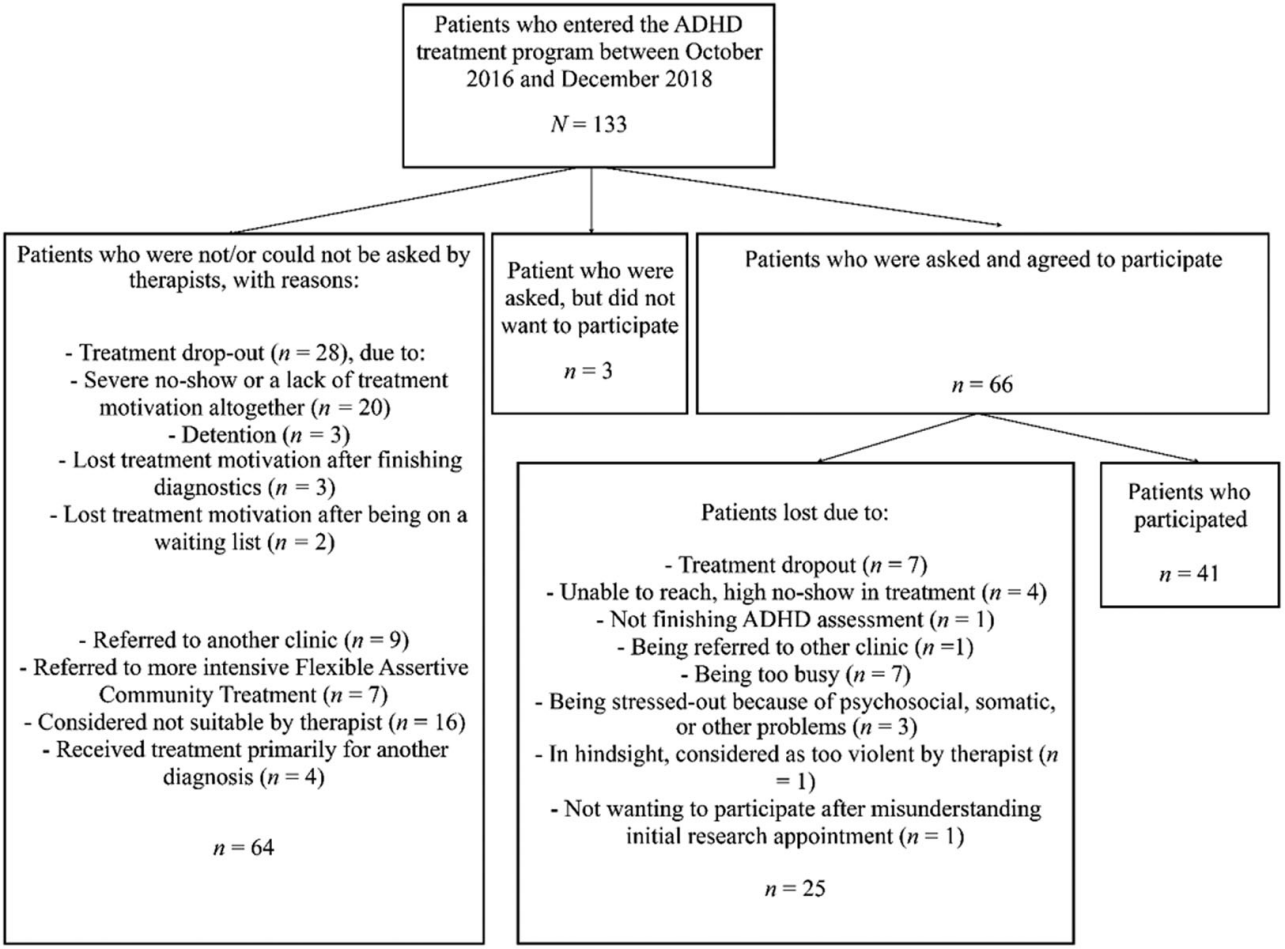
Participant inclusion in the original study

The actual research appointments took place from October 2016 until April 2019. Of the 133 patients who entered the ADHD treatment program between October 1, 2016 and December 31, 2018, we included 41 patients (30.8%). In Figure 1 participant inflow and reasons for drop‐out are reflected. In most cases, patients dropped out of treatment to early, were referred to another mental health facility, or had agreed to participate, but then dropped out of the study before the research appointment had taken place. Because this number was too small to examine multivariate associations in some of the regression models, we also made use of data collected from 11 patients during a pilot study. The pilot study was conducted in the period from January 2016 until April 2016. In contrast to the original study, in the pilot study, we also included patients who already were receiving treatment within the forensic ADHD treatment program for a longer period of time (i.e., *M* treatment duration in days = 507.09, *SD* = 674.16; range = 49–2,339). Other procedural differences included a few differences in the self‐report questionnaires used (see Section 2.3), and the fact that during the pilot study patients filled‐out the standardized questionnaires by themselves (but in the presence of one of the researchers).

### 2.3 Measures

#### 2.3.1 Cognitive‐motivational functioning

*Response inhibition* was measured with a Go/No‐Go computer task in which patients had to respond to frequent Go stimuli (the letter O; 120 trials), and inhibit responding to infrequent No‐Go stimuli (the letter X; 40 trials). The number of errors made on the No‐Go trials (i.e., errors of commission), are considered to reflect inhibitory control, with more errors indicating poorer control. The task we used was similar with regard tointertrialduration (i.e., 1600 ms), stimulus simplicity, and presentation (i.e., 200 ms) to a task used in research on adults with antisocial personality disorder (Dolan & Park, 2002) and children with ADHD (Rubia et al., 2001). Patients were instructed to press the response button as fast as they could when Go‐stimuli appeared on the computer screen, to inhibit responding to No‐Go stimuli, and to make as few mistakes as possible. Before the actual task started, patients participated in a practice block to ensure that they correctly understood the instructions. Next to the number of stopping mistakes on No‐go trials, we calculated patients' average reaction times (RT) on Go‐responses in milliseconds. Faster reaction times indicated quicker, and thus more impulsive responding (Bezdjian, Baker, Lozano, & Raine, 2009; Halperin, Wolf, Greenblatt, & Young, 1991).

*Self‐reported impulsivity* was assessed with four adjusted items (*α* = .79) of the International Personality Item Pool―Neuroticism‐Extraversion‐Openness inventory (IPIP‐NEO; Witt, Donnellan, & Blonigen, 2009). The IPIP‐NEO is originally developed to assess personality traits in the general population. Items are scored on a four‐point scale (1 = *completely disagree* to 4 = *completely agree*), with higher scores indicating higher levels of (re)acting without thinking, thus poorer inhibition:that is,“I make rash decisions,” “I jump into things without thinking,” “I rush into things,” and “I act without thinking.” Higher mean total scores indicated more impulsivity. To our knowledge, the psychometric properties of the items to assess impulsivity alone, have not yet been tested in previous research.

*Motivational functioning* was assessed with a hypothetical temporal discounting task (Scheres, Lee, & Sumiya, 2008). In this task, participants were asked to make choices between receiving smaller immediate monetary rewards and larger rewards that can be obtained later in time, based on their preferences. The amount of the delayed reward was the same in every trial (i.e., €100). The amount of the immediate rewards and the delay durations varied between trials (i.e., range €10–€100, and: 1 month, 1 year, and 5 years). This way we were able to calculate patients' temporal discounting functions. Temporal discounting refers to the fact that the subjective value (SV) of a reward decreases as the distance to the reward into the future increases. The rate at which this SV goes down as a function of waiting time varies across individuals. In experimental paradigms, temporal discounting is measured by presenting individuals with choices between a smaller immediate reward and a larger delayed reward. Typically, the immediate reward, while always smaller than the delayed reward, varies in magnitude. The delayed reward is constant in magnitude but the delay preceding its delivery varies. By analyzing the choice pattern of individuals (i.e., we calculated the proportion of delayed reward choices for each delay duration per person, multiplied this by the range of plausible SV's, and added the lowest possible SV; see Mies, Ma, De Water, Buitelaar, & Scheres, 2018), every participant gets an estimation of the SV of the delayed reward, for each delay duration. The change in SV as a function of delay duration can be plotted as a persons' discounting function (Critchfield & Kollins, 2001). The more rapidly the SV of a large reward decreases as a function of time, the steeper the discounting function and the higher the preference for immediate rewards. Based on the SV's for each delay, the “area under the curve” (AUC) of this discounting function was calculated (see for a detailed explanation: Myerson, Green, & Marusawitharana, 2001), and used as dependent variable. Smaller AUC's reflected steeper discounting and thus strong preference for smaller immediate rewards. Results of a recent meta‐analysis supported the discriminant validity of monetary temporal reward discounting tasks by showing consistent steeper temporal reward discounting in patients with ADHD compared to healthy controls (Jackson & MacKillop, 2016).

*Self‐reported motivational deficits* were assessed with the Quick Delay Questionnaire (QDQ; Clare, Helps, & Sonuga‐Barke, 2010). This questionnaire has been developed to quickly assess altered delay behavior in adults, with five items measuring delay aversion (*α* = .82) and five items measuring delay discounting (*α* = .67). Participants indicated how much (1 = *not like me at all* to 5 = *very like me*) they agreed with items, such as “Having to wait for things makes me feel stressed and tense,” and “The future is not important to me, I only consider the immediate consequences of my actions.” Higher mean total scores indicated more delay aversion and delay discounting. Previous research in adults with ADHD showed that the QDQ has sufficient internal reliability (Thorell et al., 2017). Results of that study further showed scores on the QDQ were associated with measures indicative of patients' functional impairment, but not with laboratory measures of executive functioning and discounting. This suggests that both type of measures should be used to adequately assess cognitive‐motivational functioning in patients with ADHD.

#### 2.3.2 Interpersonal functioning

*Attachment styles* were measured with the Attachment Styles Questionnaire (ASQ; Van Oudenhoven, Hofstra, & Bakker, 2003). This questionnaire assesses adult attachment from a general perspective, rather than attachment within particular relationships. Items include general statements about relationships with others, such as: “I find it relatively easy to get close to others,” and “I do not really feel safe in forming close relationships, because I fear I will get hurt.” Participants indicated on a five‐point scale (1 = *strongly disagree* to 5 = *strongly agree*) to what extent they agreed with the statements. Higher mean total scores on the attachment scales indicated higher levels of that particular attachment style. Initially four attachment style scales were computed: secure (eight items; *α* = .65), preoccupied (seven items; *α* = .84), fearful (five items; *α* = .78), and dismissive attachment (four items; *α* = .46). Yet, because the reliability of the dismissive attachment scale was insufficient in this study, we decided to calculate a combined avoidant attachment style to use in the analyses, by combining participants' mean scores on the dismissive and fearful attachment scale. The internal reliability of the combined fearful/dismissive‐avoidant style was sufficient with *α* = .70. Psychometric properties of the ASQ have previously only been tested in general populations, were the scales had sufficient reliability and construct validity (Van Oudenhoven et al., 2003).

*Social support* was assessed by asking participants to first list (a maximum of 10) social network members,that is,those members who played an important role in their lives at that moment. Next, participants were asked the following four questions (*α* = .88): “To whom of these persons you would *like* to turn to for support, in case you had a problem?”; “To whom of these persons you would *actually* turn to for support in case you had a problem?”; “On whom of these persons, you *wish* you could always count on, no matter what?”; and “On whom of these persons you can *actually* always count on, no matter what?” Social support scores were computed by summing the number of listed network members for each of the questions, and dividing this number through the total number of network members that the participant listed as playing an important role in his life at that moment. Higher scores thus indicated higher levels of (proportional) perceived social support as provided by the most important network members of each participant.

#### 2.3.3 Behavioral functioning

Externalizing behaviors were assessed with four self‐report questionnaires. A short form of the Aggression Scale (Bryant & Smith, 2001) was administered to assess self‐reported anger (three items; *α* = .69), hostility (three items; *α* = .78), and (verbal and physical) aggression (six items; *α* = .63). The Aggression Scale includes items such as “I have difficulty keeping my composure” and “Sometimes, I cannot suppress the tendency to hit someone.” Items are rated on a five‐point scale (1 = *completely disagree* to 5 = *completely agree)* with higher mean total scores indicating higher levels of externalizing behavior. The psychometric properties of the aggression scales were sufficient in previous research in (forensic) clinical samples (Hornsveld, Muris, Kraaimaat, & Meesters, 2009).

Also, antisociality was assessed with 16 items (*α* = .70) of the Impulsive Antisociality scale creation of the IPIP―NEO inventory (Wit, Donnellan, & Blonigen, 2009). Items assessing antisociality include statements such as “I take advantage of other people,” and “I obstruct other people's plans,” which were rated on a four‐point scale (1 = *completely disagree* to 4 = *completely agree*). Higher mean total scores reflected higher levels of self‐reported antisociality. During the pilot study, we administered a short‐form of the antisociality scale, including only four items (*α* = .80), which was used to compute mean total scores on antisociality in the 11 patients of the pilot.

Alcohol use was measured with four items (*α* = .77) of the Alcohol Use Disorders Identification Test (AUDIT‐4; Gual, Segura, Contel, Heather, & Colom, 2002; Saunders, Aasland, Babor, de la Fuente, & Grant, 1993), and drug use was assessed with four similar items from the Drug Use Disorders Identification Test (DUDIT; Berman, Bergman, Palmstierna, & Schlyter, 2003). The AUDIT was developed to identify risky or harmful alcohol use, and asks about people's alcohol use within the past year. In previous research, the shorter four item AUDIT‐4 detected risky drinking in clinical populations, as well as the 10‐item AUDIT, does (Gual et al., 2002). Items include: “When you drink alcoholic beverages, how often do you drink more than 6 glasses of alcohol?” and “Has a family member, friend, physician, or other professional ever worried about your alcohol consumption or given you advice to drink less?”. Participants answered questions on a five‐point scale, with higher scores indicating more severe alcohol use. The DUDIT is developed as a parallel test of the AUDIT and includes exactly the same questions but then targeted at participants' drug use. The psychometric properties of the DUDIT were satisfactory for use in clinical populations in previous research (Hildebrand, 2015).

Of note, the AUDIT and the DUDIT were not administered during the pilot study. All analyses including substance use were conducted on a smaller subsample of *n* = 41 (see also Section 2.4). In the result section, we therefore refer to ‘externalizing behaviors’ (including anger, hostility, aggression, and antisociality) and “substance use” (alcohol and drug use), as separate constructs. Yet, severe substance use is of course also externalizing behavior.

#### 2.3.4 Treatment and research engagement

No‐show on treatment appointments was tracked via electronic patient files for the first 10 appointments that were planned at the outpatient center after patients had completed data collection during the research appointment. In addition, no‐shows on research appointments were tracked by the researchers. No‐show was defined by not showing up for treatment or research, without having a reason for this that was beyond patients' control (i.e., such as having a sick child, having a death in the family, or getting into a traffic accident on their way to treatment). No‐show rates were calculated by dividing the number of no‐shows by the total number of planned appointments.

We also calculated the duration in days between the first and last of the (maximum of) 10 treatment appointments, and used this as an additional indicator of patients' treatment adherence. At the outpatient center where data collection took place, patients have generally some control over how regularly they are seen for treatment. When patients fail to show up for an appointment, some patients reschedule a new appointment as soon as possible (and therefore are considered more motivated or engaged), whereas others try to postpone rescheduling for as long as possible (and thus are considered less engaged). In addition, if applicable, treatment side modules are often provided on request. Therefore, adherent (or motivated) patients would be able to receive more (types of) treatment simultaneously, and thus generally would receive more (different types of treatment) appointments within a shorter amount of time. As such, longer treatment time intervals were reflective of poorer treatment adherence here.

#### 2.3.5 Background characteristics

Demographic information and medication use was assessed with self‐reports. Background treatment characteristics were retrieved from electronic patient files. Finally, patients' self‐reported on treatment motivation by answering the following questions on a five‐point scale (1 =* not at all* to 5 = *completely agree*): “Are you a person that is generally on time for his treatment appointment?”; “Do you consider yourself motivated for treatment at the outpatient center”; and “Do you consider the opportunity present, that you will drop‐out of treatment before all of your treatment goals are achieved”. The last question was reversely scored so that higher scores indicated higher treatment motivation. During the pilot study, only the question on being on time for treatment appointments was administered.

### 2.4 Statistical analyses

Descriptive analyses were conducted to examine score distributions and missing values. Because score distributions on self‐reported treatment motivation showed little variation between patients (i.e., almost no patients indicated not being motivated at all), scores on these variables were dichotomized into 1 = *completely motivated* (i.e., always on time, extremely motivated, and not going to dropout of treatment before all treatment goals are achieved), and 0 = *little to moderately motivated for treatment*. No‐show during the research appointments was also transformed into a dichotomized variable (1 = *having missed a research appointment* and 0 = *not having missed a research appointment*), because only three patients had missed the research appointment more than once. Eleven patients from the pilot study had missing data on two of the three self‐report questions on treatment motivation, and substance use. Because these variables could not reliably be replaced using information of the other measures administered in the study, we excluded these patients from all analyses including these variables. Five patients from the original study had missing data on one of the alcohol use questions. We tested whether these items were missing at random with Little's (1988) Missing Completely At Random (MCAR) test, and replaced the missing values by participant's mean score on the other three items measuring alcohol use (Hawthorne & Elliott, 2005). Finally, one patient from the original study dropped out of the study before finishing any of the standardized self‐report questionnaires, except for substance use. He was thus excluded from all analyses including variables assessed by the other questionnaires.

Second, we assessed whether any of the background characteristics (i.e., age, educational level, occupational status, having a judicial past, having received treatment in the past, currently receiving medication, receiving treatment as part of a judicial sentence, and self‐reported treatment motivation) were associated with no‐shows and treatment time intervals, using correlations, independent sample *t* tests, Mann‐Whitney *U* test, and chi‐square tests. Also, bivariate associations between all study variables were examined. Given the non‐normal distribution on most of the independent variables and all dependent variables, we calculated correlations with these variables using Spearman's rho. Independent sample *t* tests and Mann‐Whitney *U* tests were performed to examine whether patients with and without no‐show on research differed on any of the independent variables. Effect sizes were calculated for significant results (i.e., using Cohen's *d* for the *t* tests, and the Probability of Superiority (SP = *U*/n1 × n2) for the Mann‐Whitney *U* tests). We corrected for multiple hypotheses testing using the Holm‐Bonferroni method (Gaetano, 2013; Holm, 1979).

Third, multiple regression analyses were performed to assess associations of cognitive‐motivational, interpersonal, and behavioral functioning with treatment no‐show and treatment time intervals, while controlling for the time that patients were in treatment before the study, and the number of treatment appointments that they had planned after the study. For eight patients we were unable to follow them for 10 appointments after research participation. Four dropped out of treatment too early, and four others completed treatment successfully before this time. Logistic regression analyses were conducted to examine associations with no‐show on research. Because of the limited sample size and power issues, we tested associations for the different domains of functioning in separate analyses. Moreover, because most study variables had a non‐normal distribution, we performed bootstrapping (Russel & Dean, 2000). Multivariate outliers were checked by calculating Mahalanobis distance, Cook's, Leverage scores, and standardized residuals (>2.5; Fidell & Tabachnick, 2003), and removed if they significantly impacted the results. Cut‐off scores for possible outliers based on Mahalanobis distance were determined on the basis of the relevant chi‐square values with*p* < .001. For Cook's distance cut‐off scores were calculated using the formula: 4/(*N* − *k* − 1), and for Leverage scores using: 2*k *+ 2/*N*, where *N* is the number of cases, and k is the number of independent variables (Belsley, Kuh, & Welsch, 2005).

Finally, we conducted parsimonious regression analyses on no‐shows and treatment time intervals, including the background variables and the variables assessing cognitive, interpersonal and/or behavioral functioning that were significantly associated with the outcome variables in previous analyses. This way, we tested the robustness of our findings and examined which of the risk factors best explained variation in no‐show and treatment time intervals. Because of the missing data on substance use in patients from the pilot study, results of these final analyses are discussed separately for the total sample (*N* = 52, including patients from the pilot), and the subsample of patients (*n* = 41) without missing data. A post hoc power analysis (Faul et al., 2007) showed that we had enough power (0.82) when testing multivariate associations of large effect sizes (*f* ^2^ = 0.35) with *α* = .05, in the regression models with four predictors and *n* = 41.

## 3 RESULTS

### 3.1 Descriptive analyses and correlations

In Table 1, patients' background characteristics and descriptive information on all study variables are presented. Regarding associations with demographic and treatment background characteristics, findings showed that only age was significantly negatively correlated with no‐show on treatment (*ρ* = −0.28, *p* < .05), such that older patients had fewer no‐shows. Regarding treatment time intervals, Mann‐Whitney *U* tests indicated that patients who had received previous treatment within the general mental health system, completed the (maximum of) 10 treatment appointments within a shorter number of days (*Md* = 63.00, *n* = 35) compared to those who had no treatment history or only had received treatment within forensic care before (*Md* = 97.00, *n* = 17, *U* = 192.5, *z* = −2.05, *p* < .05, *PS *= 0.32). There were no significant differences in background characteristics between patients who had missed a research appointment and those who did not.

**Table 1:** Background and descriptive information on patients' cognitive‐motivational, interpersonal, and behavioral functioning, and treatment and research compliance (*N* = 52)

	*M* (*SD*)	Range	
Background characteristics		
Age	35.31 (9.38)	19–61	
Educational level, *n* (%)		
Low	33 (63.5)		
Moderate	19 (36.5)		
High	0 (0.0)		
Employed/full‐time study,^a^ *n* (%)	24 (46.2)		
Judicial past,^a^ *n* (%)	33 (63.5)		
Forensic treatment history,^a^ * n* (%)	16 (30.8)		
History of regular mental health treatment,^a^ *n* (%)	35 (67.3)		
Self‐reported treatment motivation			
Always on time, *n* (%)	27 (51.9)		
Fully motivated for treatment,^b^ *n* (%)	26 (63.4)		
No opportunity for early drop‐out,^b^ *n* (%)	22 (53.7)		
Cognitive‐motivational functioning			
Stopping mistakes Go/No‐Go task	11.1 (6.97)	0.00–26.00	
Reaction time go responses *ms* Go/No‐Go task	248.53 (44.43)	172.56–363.91	
Area under the curve in temporal reward discounting task	0.31 (0.24)	0.05–0.94	
Self‐reported impulsivity	2.90 (0.67)	1.50–4.00	
Self‐reported temporal discounting	2.51 (0.75)	1.00–4.40	
Self‐reported delay aversion	3.99 (0.85)	2.20–5.00	
Interpersonal functioning			
Secure attachment	3.37 (0.69)	1.71–4.71	
Preoccupied attachment	2.93 (0.99)	1.14–4.71	
Fearful/dismissive‐avoidant attachment	3.69 (0.61)	2.00–4.90	
Social support	0.53 (0.29)	0.00–1.00	
Behavioral functioning			
Anger	3.51 (1.07)	1.33–5.00	
Hostility	3.27 (1.19)	1.00–5.00	
Aggression	3.20 (0.77)	1.50–4.83	
Antisociality	2.05 (0.47)	1.00–3.50	
Substance use^b^			
Alcohol use	0.93 (0.85)	0.00–3.50	
Drug use	1.35 (1.06)	0.00–3.25	

	*M* (*SD*)	Median	Range
Treatment characteristics			
Mandatory treatment,^a^ *n* (%)	14 (26.9)		
Treatment duration at time of inclusion in days	268.71 (361.85)	148.50	49.00–2339.00
*First (maximum) 10 appointments after research participation*			
Number of treatment appointments	9.06 (2.32)		2.00–10.00
Drop‐out/treatment completed,^a^ *n* (%)	8 (15.4)		
No‐show percentage	0.24 (0.20)	0.20	0.00–0.70
Time duration intervals in days	80.17 (49.43)	72.00	7.00–242.00
Research engagement			
Number of appointments	1.5 (0.67)		1.00–3.00
No‐show,^a^ *n* (%)	17 (32.7)		

a Included dummy variables with Yes serving as the reference category.

b Eleven patients had missing data on these variables.

In Table 2, bivariate correlations between all independent variables and no‐show on treatment and treatment time intervals are presented, together with the descriptive statistics for patients who had missed a research appointment and those who did not. Patients' average RT on Go‐responses on the Go/No‐Go task was positively associated with antisociality, indicating that patients with higher levels of antisociality responded slower (i.e., less impulsive) on the Go/No‐Go task. Furthermore, self‐reported impulsivity was positively associated with avoidant attachment and externalizing behavior. Self‐reported temporal discounting and delay aversion were also negatively associated with secure attachment. Regarding interpersonal functioning, avoidant attachment was negatively associated with social support, and in general, positively associated with externalizing behaviors and drug use (i.e., *n* = 41). Externalizing behaviors were positively associated with drug use (*n* = 41). These were all small correlations.

**Table 2:** Bivariate correlations and descriptive statistics on cognitive‐motivational, interpersonal, and behavioral functioning and no‐shows and treatment time intervals (*N* = 52)

		1.	2.	3.	4.	5.	6.	7.	8.	9.	10.	11.	12.	13.	14.	15.	16.	17.	18.
1. No‐Go mistakes		–																	
2. Go RT		−.30*	–																
3. TD AUC		−.05	.29*	–															
4. Impulsivity self‐r		−.10	−.05	−.16	–														
5. TD self‐r		.15	.14	−.03	.34*	–													
6. Delay aversion		.13	.05	−.12	.31*	.58**	–												
7. Secure attach		−.00	−.15	.03	−.15	−.44*	−.45*	–											
8. Preoccupied		.13	−.04	.01	.16	.04	−.01	.20	–										
9. Avoidant		.00	.23	−.11	.33*	.47*	.54**	−.53**	−.03	–									
10. Social support		−.09	−.28	.17	−.10	−.02	.01	.20	.07	**−.29** *	–								
11. Anger		.23	.03	−.05	.32*	.32*	.44*	−.08	−.09	.39*	−.29*	–							
12. Hostility		.04	.13	.16	.21	.42*	.33*	−.11	.37*	.23	.05	.32*	–						
13. Aggression		.14	.22	.16	.22	.25	.40*	−.22	.04	.33*	.01	.57**	.28*	–					
14. Antisociality		−.06	.31*	.09	.21	.28*	.27	**−.43** *	.00	.30*	−.28*	.25	.28*	.57**	–				
15. Alcohol use		−.16	.22	−.30	−.19	−.03	−.00	.09	−.08	−.02	−.29	−.06	.14	.11	.14	–			
16. Drug use		.08	−.07	−.11	.30	.17	**.41** *	−.09	.17	.32*	−.16	.10	.42*	.27	.35*	.23	–		
17. Treatm no‐show		−.10	−.01	.01	−.08	.12	**−.31** *	.22	−.02	−.16	−.05	−.27	−.12	−.20	−.25	.25	−.22	–	
18. TreatmTime		−.01	.16	.09	**−.34** *	−.12	−.11	.12	.13	.06	−.13	.15	.17	−.17	.13	.38*	.14	0.03	–
Research no‐show														
No *n* = 34	*Md*/*M*(*SD*)	12.00	249.23 (48.40)	0.25	2.74 (0.66)	2.49 (0.76)	4.20	3.40 (0.76)	2.92	3.66 (0.61)	0.58	3.83	3.33	3.16 (0.82)	2.04 (0.49)	0.75	1.63	0.10	70.00
Yes *n* = 17	*Md*/*M*(*SD*)	8.00	247.11 (36.19)	0.31	3.22 (0.58)	2.54 (0.75)	4.00	3.31 (0.52)	3.29	3.77 (0.63)	0.37	3.67	3.67	3.26 (0.70)	2.08 (0.44)	0.50	1.25	0.33	76.00
	*U/t*	*U *= ns	*t *= ns	*U *= ns	*t* = −2.55*	*U* = ns	*U *= ns	*t* = ns	*U *= ns	*t* = ns	*U *= ns	*U *= ns	*U *= ns	*t* = ns	*t* = ns	*U *= ns	*U *= ns	*U* = 429.50*	*U *= ns

*Note*: Eleven patients had missing data on alcohol and drug use.

Abbreviations: attach, attachment; GO‐RT, reaction time in ms on go responses on the Go/No‐Go task; *M*, mean; *Md*, median; No‐Go mistakes, number of commission errors on the Go/No‐Go task; ns, not statistically significant at *p *< .05; self‐r, self‐reported; *t*, independent sample *t*‐test statistic; TD AUC, area under the curve of the temporal discounting task; treatm no‐show, no‐show rate on treatment appointments; TreatmTime, time duration in days between the first and last of maximum 10 treatment appointments; *U*, Mann–Whitney *U* test statistic.

* *p *< .05.

** *p* < .001.

Regarding associations with treatment no‐show and treatment time intervals, delay aversion was negatively associated with no‐show on treatment, and self‐reported impulsivity was negatively associated with treatment time intervals. In contrast, alcohol use was positively associated with treatment time intervals (*n* = 41). Yet, again, these correlations were small. Moreover, when controlled for multiple testing, group comparisons for no‐show on the research appointment showed that patients who had missed a research appointment reported more impulsivity than those who did not, *t*(48) = −2.55, *p* < .05; Cohen's *d* = .76). Patients with no‐show on the research appointment also had higher no‐show rates on treatment (*U* = 429.5, *z* = 2.61, *p* < .05, *PS =* 0.72). There were no significant associations between interpersonal functioning and externalizing behaviors in relationship to no‐shows and treatment time intervals.

### 3.2 Multivariate regression analyses on no‐shows and treatment time intervals

Similar to results of the univariate analyses, results of the multivariate regression analyses showed that only cognitive‐motivational functioning and substance use were significantly associated with no‐shows and treatment time intervals. As such, we only reported the results of the regression analyses including these domains of functioning in Table 3. Regarding cognitive‐motivational functioning, delay aversion was significantly negatively associated with no‐show on treatment while controlling for the time that patients had been in treatment. Patients who reported higher levels of delay aversion, thus had fewer no‐shows on treatment. In addition, self‐reported impulsivity was negatively associated with treatment time intervals, and positively with no‐show on research, such that patients with higher levels of impulsivity completed the (maximum) 10 treatment appointments in a shorter amount of time, but were more likely to have missed a research appointment. Regarding substance use (*n* = 41), alcohol use was positively associated with no‐show on treatment, and longer treatment time intervals, whereas drug use was negatively associated with no‐show on treatment.

**Table 3:** Linear and logistic regression analyses of no‐shows and treatment time intervals on cognitive‐motivational functioning and substance use

	No‐show treatment		Treatment time intervals (max) 10 appointments		No‐show research			
	*B* (SE)	95% CI	*B* (SE)	95% CI	*B* (SE)	95% CI	OR	95% CI OR
Cognitive‐motivational functioning (*N* = 52)								
Computer tasks								
*R* ^2^	0.11		0.28*		Nagelkerke *R* ^2^ = 0.37, Model *χ* ^2^ = 15.07*			
Constant	0.67 (0.23)		−62.33 (45.39)		5.32 (61.48)		205.09	
Treatment duration Incl	0.00 (0.00)	0.00; 0.00	0.03 (0.03)	−0.01; 0.11	0.00 (0.01)	0.00; 0.01	1.00	1.00; 1.01
Treatment appointments	−0.02 (0.02)	−0.06; 0.01	9.01 (1.38)*	6.24; 11.74	–	–	–	–
No‐Go mistakes	−0.00 (0.01)	−0.01; 0.01	0.52 (0.82)	−0.93; 2.40	−0.23 (2.43)*	−0.74; −0.12	0.79	0.67; 0.94
Go RT	−0.00 (0.00)	−0.00; 0.00	0.19 (0.17)	−0.10; 0.57	−0.02 (0.25)*	−0.08; −0.00	0.98	0.96; 1.00
TD AUC	0.13 (0.14)	−0.14; 0.40	3.50 (32.53)	−73.09; 56.84	1.98 (54.64)	−1.52; 6.99	7.38	0.32; 170.74
Self‐reported								
*R* ^2^	0.21		0.39**		Nagelkerke *R* ^2^ = 0.36, Model *χ* ^2^ = 15.48*			
Constant	0.78 (0.21)		65.52 (41.17)		−5.26 (3.72)		0.01	
Treatment duration Incl	0.00 (0.00)	0.00; 0.00	0.01 (0.02)	−0.03; 0.07	0.00 (0.00)	0.00; 0.01	1.00	1.00; 1.01
Treatment appointments	−0.03 (0.01)	−0.06; 0.00	8.62 (1.91)*	4.90; 12.52	–	–	–	–
Impulsivity	0.01 (0.03)	−0.06; 0.08	−31.65 (13.03)*	−56.54; −6.99	2.44 (1.32)*	1.19; 6.66	11.48	2.19, 60.27
Temporal discounting	0.05 (0.05)	−0.04; 0.14	3.11 (10.41)	−17.39; 23.66	−0.14 (0.79)	−1.86; 1.55	0.87	0.27; 2.79
Delay aversion	−0.12 (0.04)*	−0.20; −0.06	5.08 (8.37)	−11.00; 21.81	−0.84 (0.80)	−3.10; 0.17	0.43	0.13; 1.41
Substance use (*n* = 41)								
*R* ^2^	0.19		0.49**		Nagelkerke *R* ^2^ = 0.11, Model *χ* ^2^ = 3.43			
Constant	0.40 (0.15)		−34.56 (13.70)		−0.95 (1.02)			
Treatment duration Incl	0.00 (0.00)	0.00; 0.00	0.04 (0.04)	−0.01; 0.16	0.00 (0.01)	−0.00; 0.02	1.00	1.00; 1.01
Treatment appointments	−0.02 (0.02)	−0.05; 0.01	9.28 (1.47)*	6.83; 12.59	–	–	–	–
Alcohol use	0.07 (0.04)	0.00; 0.16	27.68 (8.08)*	13.25; 43.90	0.02 (0.60)	−1.23; 1.11	1.02	0.42; 2.44
Drug use	−0.06 (0.03)	−0.13; −0.00	−4.30 (5.66)	−15.65; 6.49	−0.22 (0.41)	−1.03; 0.59	0.80	0.40; 1.59

*Note*: On the basis of multivariate outliers, two patients were removed from the regression analysis of no‐show on research on computer tasks.

Abbreviations: GO‐RT, reaction time in *ms* on go responses on the Go/No‐Go task; No‐Go mistakes, number of commission errors on the Go/No‐Go task; TD AUC, area under the curve of the temporal discounting task; treatment appointments, the number of treatment appointments patients had planned within the (max) 10 appointments that no‐shows were tracked; Treatment duration Incl, treatment duration in days at the time of inclusion.

* *p *< .05;

** *p* < .001

For cognitive‐motivational functioning assessed with the computer tasks, results showed a significant association with no‐show on research after multivariate outliers of two patients (i.e., based on their increased [>2.5] standardized residuals) were removed from the data. In contrast to what we expected, stopping‐mistakes on the No/No‐Go task were negatively associated with no‐show on research, indicating that patients with more mistakes (i.e., and thus more response inhibition deficits) were less likely to have missed a research appointment. Additionally, there was a negative association between RT on Go‐responses and no‐show on research, when controlling for stopping mistakes and the time that patients had already been in treatment before participating. In contrast to the previous finding and thus in line with the expectations, this indicated that patients with longer reaction times, and therefore less impulsive responding on the Go/No‐Go task, were less likely to have missed a research appointment.

### 3.3 Parsimonious regression analyses on the total sample

Finally, we conducted multiple regression analyses on no‐shows and treatment time intervals including the background, cognitive‐motivational, and substance use variables that were significantly associated with these outcomes in previous analyses. In the total patient sample, we tested associations on treatment no‐show with delay aversion, while controlling for age. Results showed that only higher levels of self‐reported delay aversion were associated with fewer no‐show on treatment (*b* = −0.06, *SE* = 0.03, 95% CI = [−0.12; −0.01], Model *R* ^2^ = 0.14, *p* < .05). For treatment time intervals, we tested associations with having received treatment within the general mental health system in the past, and self‐reported impulsivity, while controlling for the number of treatment appointments that patients had planned during this time. Both having received treatment within the general mental health system (*b* = −26.18, *SE* = 12.36, 95% CI = [−51.52; −3.32], and having higher levels of self‐reported impulsivity (*b* = −28.28, *SE* = 8.76, 95% CI = [−44.05; −9.03], were associated with shorter treatment time intervals in days (Model *R* ^2^ = 0.43, *p* < .001). For no‐show on research, associations between self‐reported impulsivity were examined together with patients' stopping mistakes on the Go/No‐Go task, and RT on go‐responses. Results showed that in this model, only self‐reported impulsivity was significantly positively related to no‐show on research (*b* = 1.29, *SE* = 0.68, 95% CI = [0.28; 2.97], odds ratio [OR] = 3.62, OR 95% CI = [1.18; 11.11], Nagelkerke *R* ^2^ = 0.23, Model *χ* ^2^ (3) = 9.02, *p* > .05). Patients with higher levels of self‐reported impulsivity were more likely to have missed a research appointment.

### 3.4 Parsimonious regression analyses on the subsample (*n* = 41): Taking substance use into account

Finally, substance use was added to the models examining treatment no‐show and treatment time intervals in the smaller subsample, excluding patients from the pilot study. For treatment no‐show, results showed that when age, delay aversion, alcohol, and drug use were examined together, none of these variables were significantly associated with no‐show on treatment. For treatment time intervals, only alcohol use was positively related to treatment time intervals (*b* = 22.22, *SE* = 6.75, 95% CI = [7.88; 35.97], Model *R* ^2^ = 0.52, *p* < .001), suggesting that patients with more alcohol use took more time to complete the 10 treatment appointments.

## 4 DISCUSSION

In the current study, we showed that higher self‐reported impulsivity was associated with no‐show on research, and that more alcohol use was related to longer treatment time intervals in forensic patients with ADHD. In contrast, higher self‐reported delay aversion was associated with fewer no‐show in treatment. Moreover, when alcohol use was not taken into account, self‐reported impulsivity was associated with shorter treatment time intervals in a subsample of patients. Finally, neither interpersonal functioning (i.e., attachment and social support), nor any of the cognitive‐motivational functioning variables when assessed by cognitive computer tasks (and while controlling for self‐reports), were related to treatment or research engagement. These findings underline previous research pointing to externalizing behavior as a risk factor for treatment nonadherence in forensic patients with ADHD, but indicate that associations with cognitive‐motivational functioning are more complex.

In particular, our findings suggested that while the severity of patients' self‐reported impulsivity can be a risk for research no‐show, self‐reported impulsivity and delay aversion can be protective factors against treatment no‐show and longer treatment time intervals in forensic patients with ADHD. Moreover, uncontrolled for self‐reported impulsivity, response inhibition deficits seemed *less* severe in patients who had missed a research appointment. This seems to contrast studies that found cognitive‐motivational problems (in particular, response inhibition deficits measured with cognitive computer tasks) were related to poorer treatment completion in substance‐abusing and forensic patients (Fishbein et al., 2009; Vergara‐Moragues et al., 2017). The mixed results may be explained by the different treatment outcomes across studies. Delay aversion and self‐reported impulsivity may reflect patients' urgency for direct stimulation and immediate action. Therefore, these cognitive‐motivational deficits can stimulate the planning of regular treatment appointments and actually showing up, although both can still be differently associated with treatment progress. In particular because next to obvious goals of symptom relief and personal growth (Glimmerveen, Brazil, Bulten, & Maes, 2018), attending therapy provides numerous immediate rewards that may stimulate patients to show‐up, such as getting support in coping with daily problems, and having the feeling of actively working on one's problems. Of course, being present does not imply that problems are dealt with effectively.

Alternatively, it can be argued that more severe cognitive‐motivational problems in patients cause more suffering, illness awareness, and distress, which motivates patients to show up regularly (and/or enhances therapists' efforts to keep them engaged). This is particularly likely for delay aversion, as this includes patients' levels of distress and anxiousness when having to wait for things, which might also include waiting for problem diminishment, or a next treatment appointment. In previous research, illness severity (Buckalew & Buclakew, 1995), and more (acute) distress (Centorrino et al., 2001; Grunebaum et al., 1996) were also motivators for treatment adherence in other patient samples. Moreover, anxiety problems have also been associated with fewer no‐shows in a previous study on forensic patients with ADHD (Stoel et al., 2018). Together, these results suggest that more worrying and distress stimulate showing up for treatment in forensic patients with ADHD.

In explaining the contrasting findings on patients' self‐reported and computer task‐based cognitive‐motivational functioning, a few arguments are worth mentioning. First, computer tasks are conducted under highly structured circumstances, and thus may also reflect an individuals' functioning in the specific research setting and task at hand (e.g., Toplak, West, & Stanovich, 2013). In this sense, self‐reports may be closer to daily life experiences and thus have higher ecological validity. Furthermore, it is relatively hard to disentangle the exact meaning behind patients' scores on computer tasks in general. For example, the fact that the two outcome variables of the Go/No‐Go task we used to assess impulsivity (i.e., commission errors and RT on Go‐responses) led to opposite outcomes with no‐show on research, indicates at least that one of these outcomes is assessing something else. Hence, for slower RT's on Go‐responses, it can be speculated that instead of being an indicator of less behavioral impulsivity, slower responding actually reflects patients' conscious efforts to do well on the task. Instead of concluding that less impulsive patients are less likely to miss a research appointment, we should then conclude that patients with higher motivation to do well are more likely to show up during the research. Alternatively, slower RT's could indicate more attentional problems in patients with ADHD, and can result from increased RT variability in patients more generally. The latter seems to be a marker of, or a risk factor for general psychopathology (e.g., Kofler et al., 2013). Overall, these contrasting results further support previous work (Thorell et al., 2017; Toplak et al., 2014) indicating that self‐reported cognitive functioning and functioning on cognitive tasks assess different things in patients with ADHD.

Our findings on behavioral functioning are partly in line with previous research on substance abuse in other psychiatric patients (Fenger et al., 2011; Matas et al., 1992). In the current study, alcohol use was associated with longer time intervals between a fixed number of treatment appointments, and higher no‐show rates in treatment in a subsample of patients. This indicates that substance abuse is important for treatment responsivity, and in line with formal recommendations (e.g., Harris & Edlund, 2005), should receive primary attention in the beginning of treatment.

In contrast to previous research (Cullen et al., 2011; Stoel et al., 2018), next to substance use none of the other externalizing behaviors were associated with treatment and research engagement. Furthermore, patients' attachment styles and perceived social support were unrelated to treatment and research outcomes. These null findings may be due to methodological issues, such as small sample size, specificity of the sample and the study context, and some of the measurements used (see below). Moreover, patients may have perceived social support by their therapists, and therefore external (and possibly less supportive) social networks are less influential with regard to treatment planning and showing up (Skeem, Eno Louden, Manchak, Vidal, & Haddad, 2009). Unfortunately, explaining why certain of the expected findings were *not* supported by the current data is particularly difficult, and replication in a larger and more diverse (forensic) psychiatric outpatient samples is warranted before more can be concluded about the role of these factors in the treatment of forensic patients with ADHD.

Regarding demographic and treatment background factors, only a history of regular mental health care was associated with treatment adherence. One reason for this is that there was little variation in demographic factors in our study: most patients were relatively young, low educated, unemployed, had criminal history, and took some form of medication. The fact that only previous treatment within general mental health care resulted in shorter treatment time intervals in patients, may have reflected intrinsic motivation for behavioral change because of previous engagement in voluntary treatment. Previous treatment experiences may also have lowered current barriers for requesting support (Fenger et al., 2011), or can be indicative of prior learning of other effective treatment coping skills. Alternatively, patients who already had a history of treatment may have had more severe problems for which they currently received more treatments within the outpatient center. This could also have resulted in completing the 10 appointments in a shorter time.

This study had some methodological limitations. First, the small sample size, limited statistical power, and missing data on substance use, the long version of the antisociality scale and some of the self‐reported treatment motivation variables in patients from the pilot study, may have influenced the findings. Similarly, other differences in assessment procedures between the pilot and the original study may have affected the internal validity and subsequently the results of the current study. In particular, the specificity and demographic homogeneity of the sample, and the variability in the treatment they received, may have complicated finding important differentiating factors for treatment and research responsivity for adult offenders with ADHD. In future studies, it should, therefore, be aimed to further control for variability in treatment time and treatment phase between patients. Nevertheless, the current sample is a reliable representation of patients with ADHD receiving treatment within the forensic outpatient center in which the study was conducted. Second, we only included patients from one treatment program for ADHD in one forensic outpatient center in the Netherlands, which limited the generalization of our findings. For example, because of the specialized nature of this treatment program, therapists may have been particularly skilled to adjust interventions according to difficult externalizing behaviors or insecure attachment behavior, and therefore these factors were unrelated to treatment adherence. Fourth, self‐report data is subject to social desirable responding (Van de Mortel, 2008), which is particularly likely for sensitive or difficult questions filled‐out together with the researchers. Furthermore, because none of the self‐reported motivation questions regarding treatment was related to any of the treatment adherence measures, this indicates that no‐show and treatment time intervals only reflect a small part of patients' treatment engagement, and that other factors, that were not assessed, obstruct patients in behaving according to their (relatively) high motivations for behavioral change during treatment.

It can also be argued that the measure for self‐reported impulsivity was more reflective of patients' general impulsive behavior (rather than response inhibition, perse),and can be considered as an additional indicator of externalizing behavior. Also, therapist factors may have been related to variation in no‐show and treatment time intervals. Finally, we measured no‐show on research retrospectively. No‐shows on earlier treatment appointments may thus have influenced patients' engagement and their responses to the questionnaires.

Notwithstanding these limitations, some practical treatment recommendations are worth noting based on the current results. In particular, regarding the protective role of impulsivity and delay aversion in treatment planning and showing up for treatment, we want to stress that if these findings indeed can be explained by arguing that they reflect patients urge for immediacy or direct stimulation (i.e., rather than patients' levels of distress), then over time, they can become risk factors for treatment nonadherence too. Hence, high impulsive and delay aversive patients may drop out of treatment if they experience that it takes “too long” to obtain a significant result. To enhance patients' motivation throughout therapy, therapists might therefore search for constant direct rewards, and stimulating ways to shape the therapy sessions. Planning and objectifying different behavioral and psychosocial changes that help patients in obtaining their ultimate treatment goal(s), and identifying and celebrating small steps towards reaching these goals, may also stimulate adherence. Finally, another strategy to counteract delay aversive behaviors in treatment has been developed in ametacognitivetherapy for (nonoffending) adults with ADHD (Solanto et al., 2010). In this treatment, patients learn to mentalize the long‐term rewards, which they aim to obtain through therapy (or through other effortful behaviors needed to obtain long‐term goals), and visualize these when executing present behavior. This treatment strategy is intended to increase the salience of long‐term rewards, so that this can be used to stimulate active engagement in the present (Solanto, Surman, Ma, & Alvir, 2018).

## 5 CONCLUSION

In sum, the current study was the first to assess treatment adherence prospectively in forensic outpatients with ADHD, and as such, provided additional support that externalizing behavior in these patients, and alcohol use in particular, is associated with nonadherence. In addition, we showed that impulsivity can be a risk factor for no‐show on research. In contrast, because higher levels of impulsivity and delay aversion were associated with better treatment adherence, we suggested that more distress, and/or patients' need for direct stimulation can motivate forensic patients with ADHD to regularly show up. Importantly, because the subsample of patients included in the pilot study had missing data on some of the main study variables of interest (e.g., the full antisociality scale, and substance use), we were unable to conduct all analyses on the sample as a whole. Therefore, the current results should be considered preliminary. Moreover, the complexity of the various interlinked risk factors for poor functioning in the current sample may have affected the role of some of the examined risk factors. Replication in a larger, more diverse forensic psychiatric sample is warranted to test the robustness of these findings and their practical relevance. In particular, studies that allow to examine the effects of clusters of risk factors for treatment adherence in forensic patients seem important to further assist clinical practice in identifying individuals at risk for poor treatment responsivity.
